# Cryptic Variation between Species and the Basis of Hybrid Performance

**DOI:** 10.1371/journal.pbio.1000429

**Published:** 2010-07-20

**Authors:** Ulises Rosas, Nick H. Barton, Lucy Copsey, Pierre Barbier de Reuille, Enrico Coen

**Affiliations:** 1 Department of Cell and Developmental Biology, John Innes Centre, Norwich, United Kingdom; 2 Institute of Evolutionary Biology, University of Edinburgh, Edinburgh, United Kingdom; 3 IST Austria, Klosterneuburg, Austria; Duke University, United States of America

## Abstract

Crosses between closely related species give two contrasting results. One result is that species hybrids may be inferior to their parents, for example, being less fertile [1]. The other is that F1 hybrids may display superior performance (heterosis), for example with increased vigour [2]. Although various hypotheses have been proposed to account for these two aspects of hybridisation, their biological basis is still poorly understood [3]. To gain further insights into this issue, we analysed the role that variation in gene expression may play. We took a conserved trait, flower asymmetry in *Antirrhinum*, and determined the extent to which the underlying regulatory genes varied in expression among closely related species. We show that expression of both genes analysed, *CYC* and *RAD*, varies significantly between species because of *cis*-acting differences. By making a quantitative genotype-phenotype map, using a range of mutant alleles, we demonstrate that the species lie on a plateau in gene expression-morphology space, so that the variation has no detectable phenotypic effect. However, phenotypic differences can be revealed by shifting genotypes off the plateau through genetic crosses. Our results can be readily explained if genomes are free to evolve within an effectively neutral zone in gene expression space. The consequences of this drift will be negligible for individual loci, but when multiple loci across the genome are considered, we show that the variation may have significant effects on phenotype and fitness, causing a significant drift load. By considering these consequences for various gene-expression–fitness landscapes, we conclude that F1 hybrids might be expected to show increased performance with regard to conserved traits, such as basic physiology, but reduced performance with regard to others. Thus, our study provides a new way of explaining how various aspects of hybrid performance may arise through natural variation in gene activity.

## Abstract 2

Studies on natural variation in gene expression and its phenotypic effects provide fresh insights into the origins of vigour and sterility in species hybrids.

## Author Summary

A major conundrum in biology is why hybrids between species display two opposing features. On the one hand, hybrids are often more vigorous or productive than their parents, a phenomenon called hybrid vigor or hybrid superiority. On the other hand they often show reduced vigour and fertility, known as hybrid inferiority. Various theories have been proposed to account for these two aspects of hybrid performance, yet we still lack a coherent account of how these conflicting characteristics arise. To address this issue, we looked at the role that variation in gene expression between parental species may play. By measuring this variation and its effect on phenotype, we show that expression for specific genes may be free to vary during evolution within particular bounds. Although such variation may have little phenotypic effect when each locus is considered individually, the collective effect of variation across multiple genes may become highly significant. Using arguments from theoretical population genetics we show how these effects might lead to both hybrid superiority and inferiority, providing fresh insights into the age-old problem of hybrid performance.

## Introduction

Crosses between closely related species give two contrasting results [3]. One result is that species hybrids may be inferior to their parents, with reduced fertility or viability [1]. The other is that F1 hybrids may be superior (heterosis), with increased vigour [2],[4]. Hybrid inferiority is commonly explained through incompatible interactions between loci [5]–[8]. Hybrid superiority is either explained through accumulation of different recessive deleterious mutations in each species, or by loci exhibiting heterozygote advantage (overdominance) [9]. The deleterious recessives hypothesis has received support from studies on domesticated inbred varieties [10], although it is unclear how such deleterious mutations would become fixed in natural populations with larger effective population sizes (though see [11],[12]). The hypothesis of heterozygote advantage suffers from the problem that it is unclear why overdominance should be prevalent [13].

One approach to understanding the basis of hybrid performance is to analyse interspecific variation at a few interacting loci and then extrapolate these findings. Variation between closely related species mainly involves either loci with small quantitative effects, or loci conferring no detectable phenotypic effect, known as cryptic variation [14]–[20]. Gene expression studies have revealed extensive differences between species in both *cis*- and *tran*-regulation across the genome [21]–[25]. However, the relationship between such variation in gene expression and phenotype has not been extensively explored. To address this issue, we have analysed interspecific variation in expression of two interacting developmental loci in *Antirrhinum*.

The *Antirrhinum* species group of southern Europe comprises about 20 species with diverse morphologies [26],[27]. These species can be intercrossed in the laboratory to give fertile hybrids, allowing the genetic basis of the species variation to be studied. So far, these studies have largely addressed divergent traits such as flower shape and colour or leaf shape and size [28]–[32]. However, to determine whether cryptic variation may also be prevalent, we chose here to analyse a conserved trait – flower asymmetry. All the species in the group have asymmetric flowers with matching upper and lower petals. The asymmetry depends on four key dorsoventral genes, *CYCLOIDEA* (*CYC*), *DICHOTOMA* (*DICH*), *RADIALIS* (*RAD*), and *DIVARICATA* (*DIV*) [33]–[36]. *CYC* and *DICH* encode related proteins belonging to TCP family of transcription factors, whereas *RAD* and *DIV* encode members of the Myb transcription factor family. The interaction between *CYC* and *RAD* has been studied in detail [37],[38]. *RAD* is a likely downstream target of *CYC* and acts in parallel with *CYC* to control dorsal and lateral petal development. As their interaction had been well characterised, we chose this pair of genes for our studies on cryptic variation.

We show here that species of the *Antirrhinum* group exhibit variation in the levels of *CYC* and *RAD* expression. This variation is cryptic as it lies within a plateau in gene expression–morphology (GEM) space. However, phenotypic effects can be revealed by creating genotypes in which the species alleles are shifted off the plateau. By considering the consequences of such patterns of variation for multiple loci and in relation to possible gene expression–fitness (GEF) spaces, we conclude that F1 hybrids might be expected to show increased performance with regard to basic physiological traits such as growth. This finding provides an explanation for hybrid vigour that avoids some of the pitfalls of previous hypotheses. Hybrid inferiority may also be expected in the longer term for nonphysiological traits such as those involved in sexual reproduction.

## Results

### Variation in *CYC* and *RAD* Gene Expression between Species

To determine the extent of interspecific variation in *CYC* and *RAD* expression, a range of species was crossed with *A. majus*. Expression of the species allele relative to *A. majus* in the F1 hybrids was then determined by competitive reverse transcription (RT)-PCR. For this procedure, RNA was extracted from flower buds collected at the same developmental stage from individual plants (three individuals were used as replicates). The expression levels of the two alleles were distinguished by pyrosequencing [21],[39]. *CYC* and *RAD* were sequenced from each species and a region chosen that included differences between *A. majus* and the other *Antirrhinum* species. As a control, genomic DNA from the hybrids was also assayed and deviations from a 1∶1 ratio used to calculate PCR bias.

**Figure 1:**
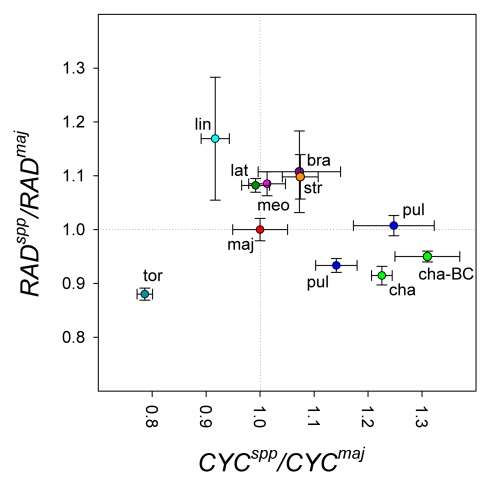
Expression of *CYC* and *RAD* gene from various species relative to *A. majus*. Significant differences in expression relative to the *A. majus* allele were observed among different species hybrids. Allele ratios in F1 hybrids with various species (spp) were obtained from competitive RT-PCR and pyrosequencing on cDNA from flower buds (stage 11). The species heterozygous with *A. majus* are: bra, *A. braun-blanquetii*; cha, *A. charidemi*; lat, *A. latifolium*; lin, *A. linkianum*; maj, *A. majus*; meo, *A. meonanthum*; pul, *A. pulverulentum*; str, *A. striatum*; tor, *A. tortuosum*. Expression levels were also obtained for heterozygotes carrying *A. charidemi* alleles in the *A. majus* background (cha-BC). Genomic DNA was used to calculate PCR amplification biases. Standard errors are shown.

Expression ratios were represented relative to alleles from the reference species *A. majus* (i.e., *CYC^maj^* = 1 and *RAD^maj^* = 1). For any comparison, the *A. majus* and other species allele will be in the same hybrid background and should thus only detect *cis*-acting differences (*trans*-acting variation should affect both alleles in the hybrid equally). Some species had alleles with significantly lower expression than *A. majus* (i.e., *CYC^tor^*, *RAD^cha^*, *RAD^pul^*), while others had higher expression levels (i.e., *CYC^pul^*, *CYC^cha^*, *RAD^lin^*, *RAD^lat^*; Figure 1). *A. pulverulentum* exhibited two different expression levels. The two expression categories correlated with different genomic DNA PCR biases and different DNA sequences, suggesting that they reflected a polymorphism within *A. pulverulentum*. Taken together, the results show that there is significant *cis*-acting variation in expression levels for *CYC* and *RAD* among the *Antirrhinum* species.

### Gene Expression and Morphology of *CYC* and *RAD* Genotypes

To determine the relationship between variation in *CYC* and *RAD* expression and developmental phenotype, a mapping between gene expression and morphology for *A. majus* was established. In what follows, we make the simplifying assumption that expression for each gene can be represented along a single axis, ignoring factors such as spatial or temporal variation in expression pattern. We also represent morphology along a single axis as this allows the GEM space to be more readily visualised. The advantage of taking such a simplified view is that it allows the key interactions and principles to be identified.

**Figure 2:**
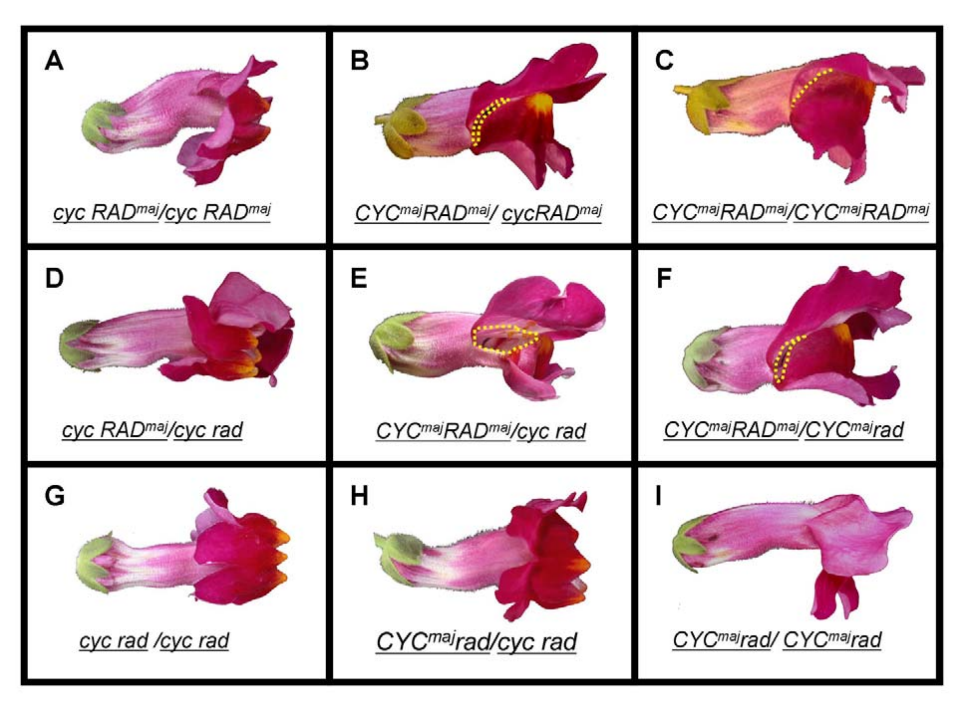
Phenotype of flowers with various *CYC* and *RAD* genotypes. Note that the double heterozygote (centre) has a notched phenotype, in which the lateral part of the flower is open. Genotypes A, B, and C were obtained by selfing a *cyc* heterozygote (genotype B). Genotypes C, F, and I were produced from selfing a *rad* heterozygote (genotype F). Genotypes C, E, and F were obtained by selfing the *cyc rad* double heterozygote (genotype E). Genotypes B and D were obtained by crossing the *cyc* mutant (genotype A) with the double heterozygote (genotype E). Genotypes F and H were obtained by crossing the *rad* mutant with the double heterozygote (genotype E). All plants were genotyped for wild-type and mutant alleles of *CYC* and *RAD*. Yellow dotted line highlights the opening between the upper and lower lobes, called “notch” phenotype.

Plants with various combinations of *CYC^maj^* and *RAD^maj^* activity were generated by crossing *A. majus* to lines carrying *cyc* and/or *rad* mutant alleles. These mutant alleles carry transposon insertions that reduce gene expression levels [33],[36]. Genotypes were confirmed using allele-specific cleaved amplified polymorphic sequences (CAPS). The resulting nine genotypes exhibited a range of phenotypes, consistent with previous studies (Figure 2) [40]. Three genotypes looked wild type (Figure 2B, 2C, 2F), as expected from the recessive nature of the mutants. The double mutant (Figure 2G) had fully ventralised flowers. Other allele combinations had intermediate phenotypes ranging from very strongly ventralised (Figure 2D, 2H), semi-ventralised (Figure 2A, 2I), to near wild-type flowers with a gap or notch between the lower and upper petals (Figure 2E).

To establish a more quantitative mapping between expression and morphology, gene expression and morphometric measurements were made for each genotype. Expression levels for *CYC* and *RAD* were determined by quantitative RT-PCR, using *UBIQUITIN* (*AmUBI*) as reference gene (Table 1). Compared to wild type, the fully ventralised *cyc rad* double mutant had *CYC* and *RAD* expression levels of less than 1% (Table 1, row G). In single *rad* homozygotes (Table 1, row I) expression of *RAD* was less than 0.14% of wild type, whereas *CYC* expression remained unaffected. In single *cyc* homozygotes (Table 1, row A), expression of *CYC* was down to 1%, whereas *RAD* was reduced to 20%. The reduced expression of *RAD* in these plants was consistent with *RAD* being a downstream transcriptional target of *CYC*. The residual *RAD* expression of 20% was presumably driven mainly by *DICH*, which acts redundantly with *CYC* [34]. Single heterozygotes for *CYC* (Table 1, row B) or *RAD* (Table 1, row F) showed about 50% expression of the relevant gene, indicating that there was little dosage compensation. The other genotypes gave further combinations of expression levels. Taken together the genotypes defined nine positions in *CYC-RAD* expression space.

**Table 1:** Gene expression and morphology for *CYC* and *RAD* genotypes.

Row	Genotype	*CYC* expression	*RAD* expression	*DI*_cor_
A	*cyc RAD^maj^*/*cyc RAD^maj^* (*n* = 4)	0.01±1×10^−2^	0.21±0.01	0.35±0.04
B	*CYC^maj^ RAD^maj^*/*cyc RAD^maj^* (*n* = 10)	0.47±0.03	0.70±0.08	0.91±0.02
C	*CYC^maj^ RAD^maj^*/*CYC^maj^ RAD^maj^* (*n* = 8)	1.00±0.09	1.00±0.08	1.00±0.01
D	*cyc RAD^maj^*/*cyc rad* (*n* = 6)	1.42×10^−3^±2×10^−4^	0.05±1×10^−3^	0.36±0.02
E	*CYC^maj^ RAD^maj^*/*cyc rad* (*n* = 14)	0.61±0.03	0.51±0.02	0.76±0.03
F	*CYC^maj^ RAD^maj^*/*CYC^maj^ rad* (*n* = 10)	0.82±0.11	0.38±0.04	0.87±0.03
G	*cyc rad*/*cyc rad* (*n* = 8)	8.30×10^−3^±1×10^−3^	1.28×10^−6^±7×10^−7^	0.0±0.01
H	*CYC^maj^ rad*/*cyc rad* (*n* = 6)	0.30±0.03	1.30×10^−5^±7×10^−6^	0.28±0.04
I	*CYC^maj^ rad*/*CYC^maj^ rad* (*n* = 4)	1.17±0.14	1.58×10^−6^±2×10^−7^	0.39±0.01

**Figure 3:**
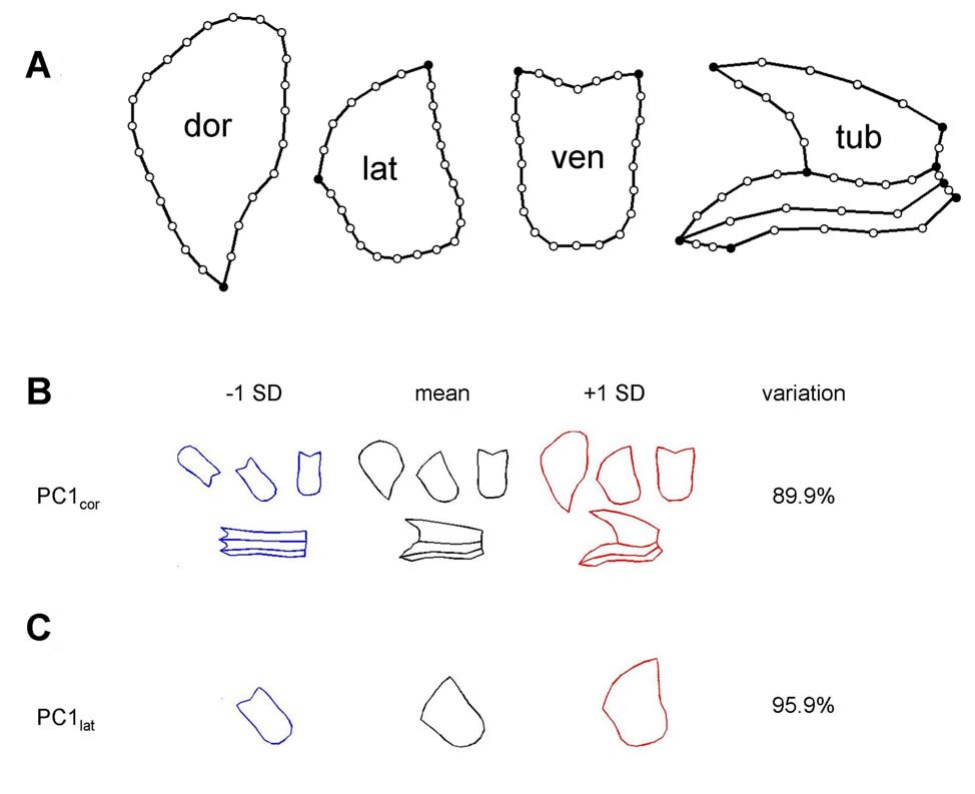
Petal outlines and principal components used to define a dorsalisation index. (A) The corolla template comprised 112 landmarks (primary landmarks in black), placed on the outlines of dorsal (dor), lateral (lat), and ventral (ven) lobes and on half of the tube (tub). (B) Effect of varying PC1_cor_ values by ±1 standard deviation (SD) about the mean. The PC1_cor_ value of −1 SD gives petal shapes similar to a fully ventralised flower while a value of +1 SD gives petal shapes more like wild type. (C) Effect of varying PC1_lat_ by ±1 SD about the mean. PC1_lat_ was based on variation in a 25-landmark template of the lateral lobe.

To allow a GEM space to be visualised, a single morphometric measure was needed for each genotype. To obtain this measure, the corolla was first dissected and flattened. 112 points were then placed around the petal outlines to capture their overall shape and size (Figure 3A). Some of these points (primary landmarks) were placed at recognisable features such as petal junctions, whereas others (secondary landmarks) were regularly spaced between the primary landmarks. This procedure was followed for eight wild-type and eight fully ventralised (*cyc rad*/*cyc rad*) flowers (alleles that are linked in coupling, i.e. are on the same chromosome, are shown underlined). The resulting 16 sets of coordinates were aligned (Procrustes alignment) and subjected to principal component analysis (PCA). A statistical model was obtained yielding one PC that captured most of the variation (90%) between the wild-type and the ventralised flower phenotypes (Figure 3B). For convenience, the PC values were scaled such that the mean ventralised mutant had a value of 0 and the mean wild-type a value of 1. The PC could therefore be considered as a dorsalisation index (*DI*_cor_) that provided a quantitative measure of variation in corolla morphology. Projection of the wild-type and fully ventralised petals onto *DI*_cor_ yielded two distinctive groups, separated according to phenotype.

The *DI*_cor_ for each of the nine genotypes was determined by flattening their petals, placing landmarks, and projecting their coordinates onto *DI*_cor_, which revealed that all genotypes had a *DI*_cor_ between 0 and 1 (Table 1). The single heterozygotes (Table 1, rows B and F) had a *DI*_cor_ of slightly less than 1. This difference from wild type was reproducible and observed in families segregating for the alleles. This finding indicates that the mutants are not fully recessive when assayed by this quantitative measure. The remaining genotypes gave lower *DI*_cor_ values, reflecting their degree of ventralisation.

### GEM Space

**Figure 4:**
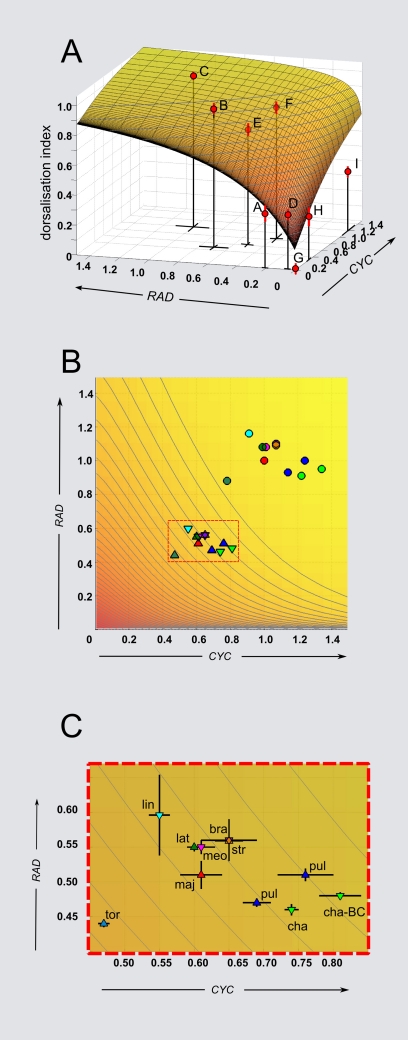
GEM spaces for *CYC* and *RAD*, showing location of various genotypes and species. (A) Dorsalisation index for each position in GEM space using values from Table 1. Standard errors for *DI*_cor_ and expression levels are shown (if error bars are not visible, they are smaller than the symbols). A smooth surface has been fitted to the data (see Materials and Methods for details of surface fitting). Note that the wild-type, C, lies on a plateau while the double heterozygote, E, is on the slope. (B) Top view of the GEM space, incorporating the relative expression values from the species taken from Figure 1 (circles). These values were adjusted assuming that *A. majus* (red circle) is at position (1, 1) in gene expression space. Triangles indicate expected gene activity values in the double heterozygote (*CYC* = x×0.6; *RAD* = y×0.5; see Table 1E). Some of the double heterozygotes are predicted to have *DI* values above or below the position of *A. majus*. Triangles pointing upwards indicate species showing notch phenotype. (C) Enlargement of rectangle in (B). bra, *A. braun-blanquetii*; cha, *A. charidemi*; lat, *A. latifolium*; lin, *A. linkianum*; maj, *A. majus*; meo, *A. meonanthum*; pul, *A. pulverulentum*; str, *A. striatum*; tor, *A. tortuosum*; cha-BC, introgression of *A. charidemi* into *A. majus* background.

A GEM space was constructed by plotting *DI*_cor_ for each genotype against its gene expression levels for *CYC* and *RAD* (Figure 4A). To get a better impression of the shape of the space, a continuous function was used to capture the main trends of the observed values. The resulting smooth GEM space gave a *DI*_cor_ that climbed from where values of *CYC* and *RAD* expression were low to a plateau where gene expression was high. Plotting the gene expression levels for the species relative to wild-type *A. majus* (coordinates *CYC* = 1, *RAD* = 1) within the same space showed that they were all located on the plateau of high *DI*_cor_ (Figure 4B). This finding is consistent with all species having asymmetric and fully closed flowers. Thus, even though there is variation in expression between species, the variation is cryptic at the morphological level because of the plateau in GEM space.

### Introgression Analysis of *A. charidemi*

One way of revealing the cryptic variation would be to shift the species off the plateau onto a steeper part of the GEM space by creating double heterozygotes. In *A. majus* double heterozygotes, gene expression levels are shifted to position (*CYC*≈0.6, *RAD*≈0.5), which corresponds to a *DI*_cor_ of 0.76 and lies just below the plateau in GEM space. If a similar shift is applied to the species, several distinct *DI*_cor_ values would be expected as the species are unlikely to fall on exactly the same *DI*_cor_ contour as *A. majus* (Figure 4B and 4C). Because *DI*_cor_ can only be strictly determined within the *A. majus* background, testing this prediction would require alleles from the species to be introgressed into the *A. majus* background followed by creation of the double heterozygotes. As an introgression programme was already underway for *A. charidemi*, this species was chosen for further analysis.

**Figure 5:**
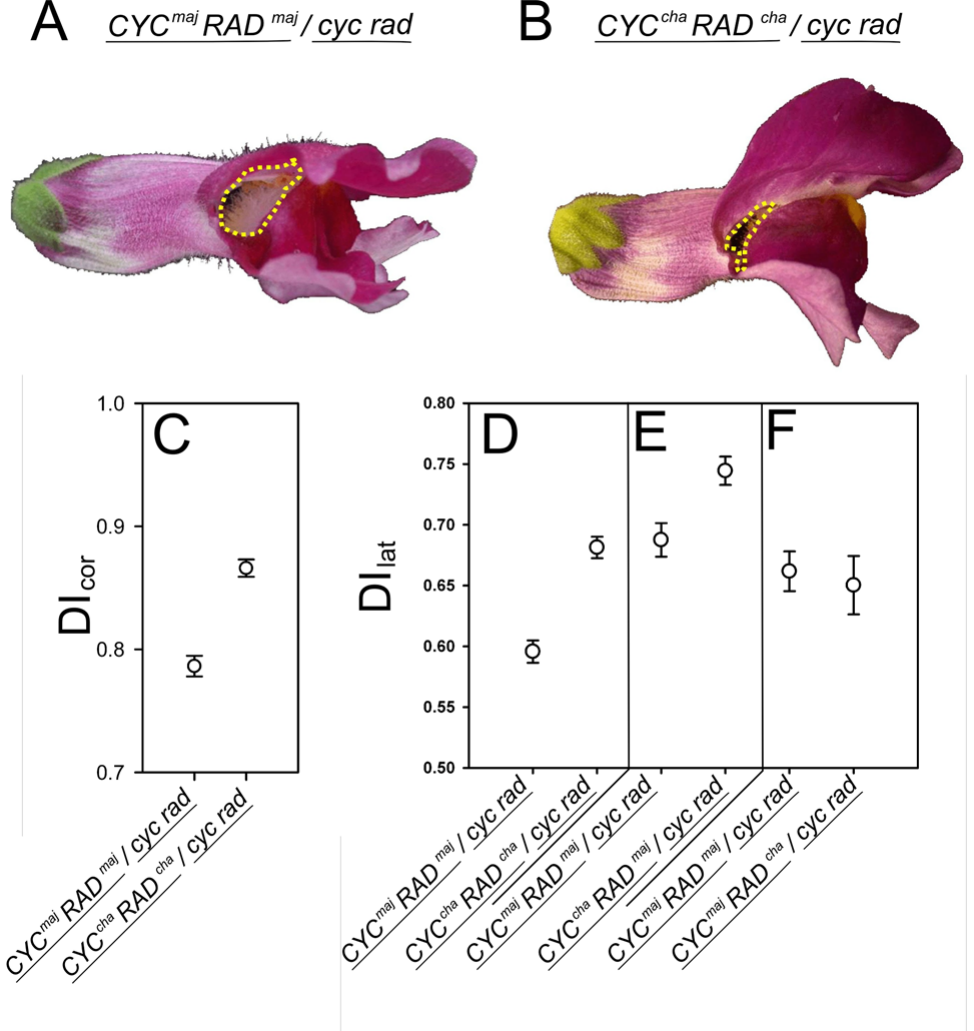
Phenotypes and *DI* values for introgressed lines. (A and B) Segregating phenotypes obtained from crossing *CYC^maj^RAD^maj^*/*CYC^cha^RAD^cha^* to the double mutant *cyc rad/cyc rad*. Yellow dotted line shows where the rims of the lateral and dorsal tube meet and highlights the difference between the effect of *A. charidemi* alleles (left) and those of *A. majus* (right). (C) *DI*_cor_ values for the genotypes in (A and B). Note that the *A. charidemi* alleles give a higher *DI*_cor_ value than the *A. majus* alleles (two-sided *t*-test _114.87_df = 7.24, *p*<0.001). (D) *DI*_lat_ values for progeny obtained from *CYC^maj^RAD^maj^*/*CYC^cha^RAD^cha^* crossed to the double mutant *cyc rad/cyc rad*. The *A. charidemi* alleles give a higher *DI*_lat_ value than the *A. majus* alleles (two-sided unpaired *t*-test _508_df = 6.73, *p*<0.001). (E) *DI*_lat_ values for progeny from a plant heterozygous for a recombinant allele (*CYC^maj^RAD^maj^*/*CYC^cha^RAD^maj^*) crossed to the double mutant *cyc rad/cyc rad*. Plants carrying the *CYC^cha^* allele have a higher *DI*_lat_ value than those carrying the *CYC^maj^* (two-sided *t*-test _202.37_df = 3.14, *p* = 0.002). (F) *DI*_lat_ values for progeny from a plant heterozygous for a recombinant allele (*CYC^maj^RAD^maj^*/*CYC^maj^RAD^cha^*) crossed to the double mutant *cyc rad/cyc rad*. Plants carrying the *RAD^cha^* allele are not significantly different from those carrying *RAD^maj^* (two-sided *t*-test _202.37_df = 3.14, *p* = 0.002). Bars indicate standard errors.

Using CAPS and amplified fragment length polymorphism (AFLP) markers *CYC* and *RAD* alleles from *A. charidemi* were introgressed into the *A. majus* background. At the backcross 5 (BC5) generation, plants with genotype *CYC^maj^RAD^maj^/CYC^cha^RAD^cha^* were crossed to the double mutant *cyc rad*/*cyc rad* generating two main genotypes. *CYC^maj^RAD^maj^/cyc rad* showed the expected notched morphology corresponding to a *DI*_cor_ of 0.78 (Figure 5A, 16 plants). By contrast, *CYC^cha^RAD^cha^*/*cyc rad* had a morphology more similar to wild type (Figure 5B) and had a significantly higher *DI*_cor_ of 0.86 (Figure 5C, 11 plants). This finding indicates that the previously observed expression difference between *A. charidemi* and *A. majus* alleles had a phenotypic effect in a double heterozygous background.

To confirm that this effect was significant, a larger population was analysed (131 plants). Rather than using the entire corolla for calculating the *DI*, only the lateral lobe was used as this could be processed more readily. A new *DI* index, *DI*_lat_, was constructed by placing 25 points around the lateral lobe of wild-type and ventralised mutant flowers, capturing 96% of the variation (Figure 3C). This *DI* index was shown to be strongly correlated with the *DI*_cor_ index for flowers in which both were determined (Pearson product moment correlation *R* = 0.91, *p*<0.001). *CYC^cha^ RAD^cha^/cyc rad* (69 plants) had a *DI*_lat_ value of 0.68, which was significantly higher than the *DI*_lat_ value of 0.59 for *CYC^maj^ RAD^maj^/cyc rad* (63 plants) (Figure 5D). These results confirm that alleles from *A. charidemi* confer greater dorsalisation than those from *A. majus* in a doubly heterozygous background.

To determine the individual contribution of *CYC* and *RAD* to the observed difference in *DI*_lat_, recombinant *CYC^cha^ RAD^maj^* and *CYC^maj^ RAD^cha^* chromosomes were obtained by screening BC5 progeny with CAPS markers (*CYC* and *RAD* are 3cM apart [41]). The recombinants were crossed to the double mutant *cyc rad*. *CYC^cha^ RAD^maj^/cyc rad* had a *DI*_lat_ of 0.74, greater than *CYC^maj^ RAD^maj^/cyc rad*, whereas *CYC^maj^ RAD^maj^/cyc rad* had a *DI*_lat_ of 0.68 similar to *CYC^maj^ RAD^maj^/cyc rad* (Figure 5E). This indicates that the shift in *DI*_lat_ between *A. majus* and *A. charidemi* mainly reflects a change in *CYC* activity, consistent with the observed higher levels of *CYC^cha^* expression in *A. majus/A. charidemi* F1 hybrids (Figure 1).

**Figure 6:**
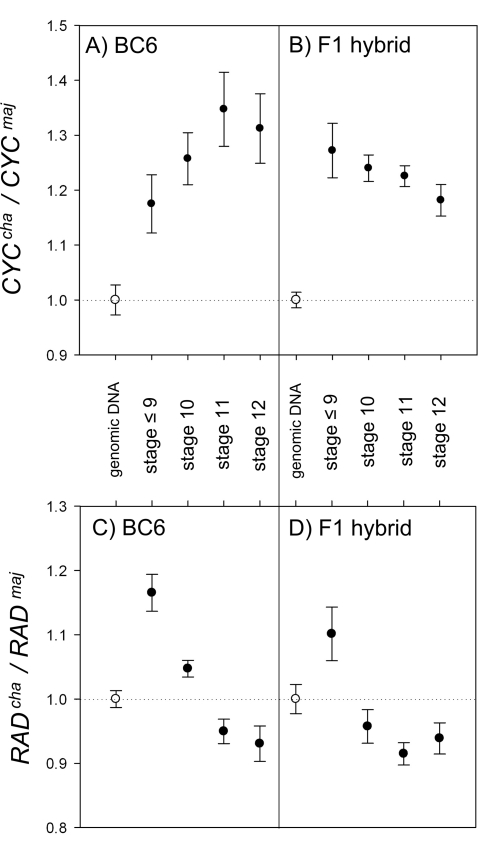
Expression ratios of *A. charidemi* alleles in various genetic backgrounds. (A and B) Ratios of *CYC^cha^* expression relative to *CYC^maj^* for BC6 (A) and F1 hybrids (B) at various developmental stages. The *CYC^cha^* allele is expressed at a higher level than *CYC^maj^* at all stages. (C and D) Ratios of *RAD^cha^* expression relative to *RAD^maj^* for BC6 (A) and F1 hybrids (B) at various developmental stages. *RAD^cha^* expression was higher than *RAD^maj^* only at the earliest developmental stage analysed. Genomic DNA (white circles) was used to calculate PCR amplification biases. Bars, standard error.

To confirm that the differences in gene expression were maintained in the *A. majus* background, allele expression was compared in the introgression lines. Consistent with the expression analysis on the F1 hybrid, expression of *CYC^cha^* was about 30% higher than that of *CYC^maj^* in BC6 *CYC^maj^RAD^maj^/CYC^cha^RAD^cha^* flower buds (Figure 6A, stage 11). This finding confirmed that the variation in *CYC* expression observed in the F1 hybrid was due to *cis*-regulatory differences. There was no significant difference between expression of *RAD^cha^* and *RAD^maj^* (Figure 6C, stage 11), irrespective of whether plants carried *CYC^cha^* or *CYC^maj^* (unpublished data). Thus, the position of *A. charidemi* in gene expression space was very similar for the F1 and BC6 plants (Figure 4B, 4C).

Expression analysis was also carried out at various developmental stages for F1 and BC6 plants to determine whether the relative expression levels were maintained. At all stages tested, expression of *CYC^cha^* was about 30% higher than that of *CYC^maj^* in F1 or BC6 plants (Figure 6A, 6B). Expression of *RAD^cha^* was also found to be higher than that of *RAD^maj^* but this difference was only observed at earlier developmental stages (Figure 6C, 6D). The early enhancement of *RAD^cha^* was observed irrespective of whether plants carried *CYC^cha^* or *CYC^maj^* (unpublished data). This difference in *RAD* expression appears to make little contribution to the phenotype because as previously shown, *CYC^maj^ RAD^cha^/cyc rad* had a similar *DI*_cor_ to *CYC^maj^ RAD^maj^/cyc rad* (Figure 5F).

Given the similarity between the results obtained for F1 and introgressed *A. majus* backgrounds, double heterozygotes with further *Antirrhinum* species were generated by crossing them to *cyc rad*/*cyc rad* plants of *A. majus*. A strong notch was observed in flowers of hybrids with *A. tortuosum* (Figure S1), as might be expected from its relatively low levels of *CYC* and *RAD* expression (Figure 1) and predicted position in GEM space (Figure 4C). A mild notch was observed with *A. latifolium* and no notch with *A. braun-blanquetii*, again consistent with their position in GEM space. However, a notch was observed in *A. pulverulentum* even though its *CYC^pul^* expression was higher than *CYC^maj^*. This difference from expectation may reflect alterations in timing of expression or contribution of other factors in the genetic background of these F1s.

### GEF Space

The observed pattern of interspecific variation most likely reflects the interaction between gene expression and fitness. This relationship can be represented by a GEF space, which is similar to GEM space except that fitness instead of morphology is plotted on the vertical axis. As with GEM space, we make the simplifying assumption that gene expression for each gene can be represented as a single axis, ignoring variation in expression pattern (which would correspond to further axes). GEF space is related to GEM space because fitness depends on how particular morphologies influences survival and reproduction. However, GEF and GEM spaces are unlikely to have precisely the same form because a small change in morphology may have a dramatic effect on fitness, and because morphologies assessed in the laboratory may not be precisely the same as those found in nature. To account for the observed pattern of variation in *CYC* and *RAD* expression, we consider various possibilities for GEF space.

**Figure 7:**
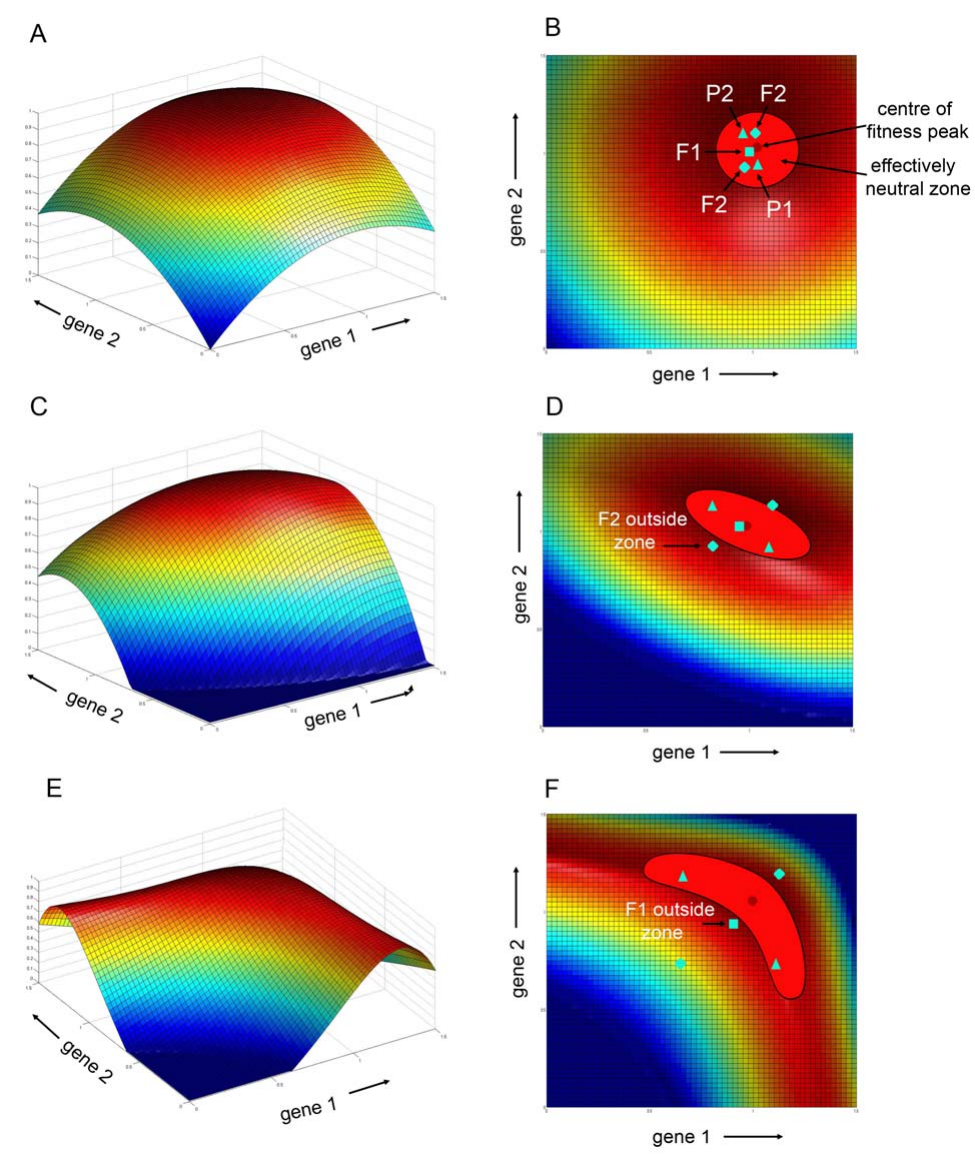
GEF spaces. Gene expression levels for two genes are plotted along the horizontal plane while fitness is along the vertical axis. (A) Radially symmetrical peak. (B) 2-D Projection of (A) showing location of effectively neutral zone and position of two parental genotypes (P1, P2 triangles), the resulting F1 (square) and additional genotypes observed in the F2 (diamonds). The F1 in this case is nearer to the centre of the peak while the F2s have similar fitness to the parents. (C) Diagonal ridge. (D) 2-D projection of diagonal ridge showing tilted elliptical neutral zone. The F1 is nearer to the peak than the parents but some F2 genotypes may now have lower fitness and fall outside the neutral zone. (E) Curved ridge. (F) 2-D projection of curved ridge showing banana-shaped neutral zone. Some F1 genotypes may have lower fitness and fall outside the neutral zone.

If we assume that GEF space has a peak around the centre of our observed expression values (Figure 7A), then there will be a zone around the peak in which variation will be effectively neutral. The extent of the neutral zone will depend on the shape of the peak and the effective population size *N_e_*. If *N_e_* = 500, then the neutral zone will occupy a region with fitness values ranging from 1 at the centre of the peak to about 0.999 (i.e., 1−1/2*N_e_* = 0.001) [42] at its rim. For a radially symmetrical peak, this zone would form a circular domain in gene expression space (Figure 7B). Gene expression values would be expected to drift within this domain, accounting for the clustered distribution of observed expression levels.

This random drift away from the optimum would reduce mean fitness, generating a “drift load.” For a wide range of models, this load is ∼1/4*N*_e_ for each degree of freedom (df), and is independent of the selection strength (see Materials and Methods). This load would have little impact on a moderately large population for one or two loci. However, if this scenario applies to many loci, say n≈1,000, then the fitness cost could be substantial. Under this scenario, F1 hybrids would gain a major fitness benefit, because each species would represent a different sample of gene expression space around the fitness peak. An F1 would represent the mean of two samples (assuming that gene expression shows additive inheritance) and is therefore likely to be nearer the peak than each individual sample (see illustrated genotypes in Figure 7B). More precisely, the variance around the optimum of the mean of two independent populations is half that of either one, and so the “drift load” is half as great (i.e., 1/8*N*_e_ per degree of freedom). For example, with 1,000 loci and an effective population size of 1,000, the fitness benefit would be 0.125, which is very substantial (i.e., 1,000×1/(8×1,000)).

This major fitness benefit would break down in the F2, as genes segregate to give a fitness for each offspring that is the same, on average, as that of the parents. If variation in expression for each locus is determined by *k trans*-acting factors with similar effects, then the F2 variance is δ^2^/(8*k*), where δ is the difference between parental means [43]. However, there may be substantial cryptic variation, with divergence due to alleles acting in opposite directions, which can produce a high F2 variance, and strong dysgenesis (see [44]). If variation is determined by *cis*-acting differences, as described here, then the F2 breakdown follows Mendelian segregation. Under this scenario with radially symmetrical peaks, or peaks that are elongated parallel to one or other axis of GEF space (i.e., ridges with elliptical neutral zones oriented parallel to the gene axes; Figure 7B), the F2 is spread over a region bounded by the parental values, and so the fitness is intermediate between the F1 and the parentals.

Another scenario is that if the peak in GEF space is elongated in a direction that is tilted with respect to the gene expression axes (Figure 7C), then, the effectively neutral zone of such ridges would form a tilted ellipse (Figure 7D). This scenario seems the most likely for the *CYC* and *RAD* genes, as it matches the orientation of the plateau edge in GEM space and also matches the distribution of gene expression coordinates for most of the species. When applied to multiple pairs of loci, the consequence of tilted neutral zones for F1 fitness would be similar to that for untilted zones described above – F1s would be expected to be nearer to the centre of the peak and have half the drift load as the parents. However, in contrast to the untilted zones, the fitness of many F2 progeny would be expected to be lower than for the parents. This lower fitness is because segregation in the F2 would lead to many gene combinations ending up on the more steeply declining slopes and thus lying below the parents in fitness (see example in Figure 7D).

Finally, we consider a scenario in which the region of high fitness is curved in GEF space (Figure 7E). In this case, the effectively neutral zone forms a banana shape. F1 hybrids between genotypes lying at different ends of the banana would have lower fitness than the parents because they would fall in the groove of the fitness surface (Figure 7F). Such loci would therefore lead to dysgenic effects in both the F1 and F2. In its extreme form, when the neutral zone is bent to form an L-shape, the fitness distribution corresponds to Dobzhansky-Muller incompatibility.

Our use of a GEM landscape is similar to Rice's framework, which maps phenotype onto a set of developmental characters [45], in our case, gene expression. Rice [45] shows how stabilising selection on the phenotype can lead to canalisation, such that the phenotype tends to be buffered against fluctuations in the underlying traits. This finding has much in common with the evolution of dominance, where buffering can evolve in a similar way. The process is driven by selection to reduce the variance of the trait, which we do not consider here. Also, “characters” may themselves be polygenic traits, whereas we focus on *cis*-acting variation at single genes. Despite these differences, there are intriguing parallels that would reward further study.

## Discussion

Species from the *Antirrhinum* group exhibit significant *cis*-acting variation in levels of *CYC* and *RAD* expression. This variation is cryptic, having no obvious phenotypic consequences in a wild-type genetic background. The lack of phenotype arises because the species lies on a plateau in GEM space. However, phenotypic effects can be revealed if the species are shifted off the plateau by construction of double heterozygotes. In such backgrounds, species carrying alleles with relative high levels of gene expression exhibit near wild-type phenotypes, whereas species with lower expression tend to give notched flowers with reduced dorsalisation. Recombination analysis allows the contribution of each locus to be determined. In the case of *A. charidemi*, the main source of phenotypic variation is due to a difference in expression at the *CYC* locus.

Our findings bridge those from several other studies. Comparative analysis of *Caenorhabditis* species has revealed changes in genes controlling the conserved trait of vulval development [18]. These changes are more substantial than those we describe, most likely because of the different timescale of these studies: divergence times are about 14–18 million y for *Caenorhabditis* species [46], compared to 1–5 million y for *Antirrhinum* [47]. Cryptic variation underlying vulval development has also been described within *Caenorhabditis* species [20], although the genetic basis of this variation has yet to be determined. Extensive variation in gene expression has been observed between *Drosophila* sibling species [21],[22] and its phenotypic consequences studied for divergent traits, such as denticle pattern [48]–[50]. Divergent ecological traits have also been shown to reflect variation in gene activity within *Arabidopsis thaliana* [51]–[54]. Our results suggest that variation in gene expression may also be found to underlie highly conserved traits and that this variation can have phenotypic consequences in certain genetic backgrounds, depending on the structure of the GEM spaces involved.

Although cryptic variation of the kind we describe would be expected to have little effect on species hybrid performance for each locus, we show that the cumulative effect of such variation at many loci could have a major effect. The magnitude and direction of this effect depends on the population size and topography of the GEF spaces in the various species habitats. For example, if GEF spaces have a radial fitness peak that is preserved across habitats, then for 1,000 loci and an effective population size *N*_e_ of 1,000 the fitness benefit in species hybrids would be 0.125, which is very substantial. This benefit arises because each species is expected to drift around its fitness peak within a radial neutral zone. For multiple loci this causes each species to lie significantly below the optimum (i.e., there is a drift load). As the species diverge, they come to represent separate samples of the neutral zone, each carrying a different combination of alleles contributing to drift load. A species hybrid will then represent an average of two samples and is therefore expected to lie nearer the peak than either sample alone, creating hybrid superiority. This hybrid benefit will be lost in the F2 as the alleles segregate, creating genotypes with fitness similar to those of the parents.

GEF spaces with elongated peaks and elliptical neutral zones are also expected to show similar benefits in hybrid fitness. However, in this case the F2 genotypes are expected to have lower fitness than the parents when the elliptical zones are tilted in gene expression space. Hybrid inferiority arises for GEF spaces that have curved or L-shaped neutral zones (L-shaped zones correspond to the standard Dobzhansky-Muller incompatibility).

The phenotype and fitness of species hybrids will reflect the extent to which these various GEF scenarios apply to the many thousands of genes in the genome. Radial or elliptical neutral domains, centred around a common position in GEF space, would be expected for loci that are under similar normalising selection in multiple environments. This situation likely applies to the *CYC* and *RAD* genes as all species in the *Antirrhinum* group have similar asymmetric closed flowers. It would also be expected for many loci controlling basic physiology and growth. F1 hybrids would therefore be expected to show higher fitness and increased performance with respect to these traits. This provides an explanation for hybrid vigour that avoids the pitfalls of previous models that require fixation of loci with major deleterious effects or that invoke special mechanisms for heterozygote advantage. A similar explanation has been proposed to account for the origin of hybrid vigour between domesticated inbred lines [10]. Hybrid vigour is usually lost in F2s or recombinant inbred lines, indicating that many of the loci involved interact to give tilted rather than untilted neutral zones.

Although hybrid vigour is commonly observed for physiological traits, the overall fitness of species hybrids is often lower than that of the parents, with sterility or other dysgenic effects being observed. This observation may partly reflect adaptation to different environments and thus shifts in the shape of fitness surfaces that drive changes in genotype. However, it may also reflect loci that interact to give curved or L-shaped neutral zones [8]. Such zones will be prevalent for traits that involve more complicated epistatic interactions, perhaps accounting for the dysgenic effects observed in F1s. The negative contribution of loci with curved neutral zones is likely to increase with time, as loci drift towards the extremities of the banana-shaped neutral domains.

The overall fitness of an F1 hybrid will depend on the relative contribution of superior and inferior effects across the genome. In this paper we have concentrated on variation in gene expression levels. Other forms of variation, such as in gene expression patterns, protein activities, or chromosome arrangements are also likely to play an important role in species divergence. The corresponding fitness spaces may be more difficult to visualise because variation for each gene may no longer be represented along a single axis. Nevertheless, the principles may be similar to those described above, with both hybrid inferiority and superiority reflecting effectively neutral variation at multiple loci, but differing with respect to the topography of the fitness spaces involved.

## Materials and Methods

### Gene Expression Analyses

Total RNA was extracted using RNeasy Plant Mini-kit (Qiagen). Total RNA was treated with DNaseI Amplification Grade (Invitrogen), and cDNA synthesized using Superscript III (Invitrogen), priming with oligo dT. Genomic DNA contamination was verified using the oligos tatgtaatttcactttaatttcgtctg and tgcttcgtttattatctgaacgatt spanning from the intron towards the 3′UTR in *RAD* (annealing temperature 55°C). Absence of a 1,010-bp PCR product after 30 cycles was considered as evidence of genomic DNA-free cDNA samples.

For expression analysis with competitive RT-PCR and quantitative sequencing, standard procedures were followed [21],[39]. For *CYC*, an assay was designed to detect a polymorphism G/A conserved in *CYC* sequences. *CYC* competitive PCR was done with the oligos [5′Btn]gcagcagccaaagagtcgag and cctgctgatgaaacccgaaaa, giving a PCR product of 172 bp, and the sequencing oligo aacaaacgcctcacg. *RAD* sequences (∼1,452 bp) from species were obtained using the oligos tccaacaagaccttttgattcc and tgcttcgtttattatctgaacgatt, spanning from the 5′UTR towards the 3′UTR, including the two exons and the intron. Sequences were aligned to the *RAD^maj^* (GenBank AY954971 http://www.ncbi.nlm.nih.gov/nuccore/61652984), and a conserved G/A polymorphism was identified. An assay was designed using the oligos aagtccgccaaggagaacaaa and [5′Btn]acggccctagccacgtta giving a PCR product of 89 bp, and the sequencing oligo ccaaggagaacaaagc. For both assays, competitive PCRs were done with an annealing temperature of 55°C to saturation (55 cycles). Genomic DNA from every plant was also included as control for allele-specific PCR biases. All PCR reactions were done in quadruplicate. For pyrosequencing sample preparation was done using the PSQ-kit (Biotage), and quantitative sequencing in a PSQ-96 sequencer (Biotage).

For quantitative RT-PCR, *AmUbiquitin* (*AmUBI* GenBank X67957 http://www.ncbi.nlm.nih.gov/nuccore/16070) was used as reference gene. Oligos were designed to flank both sides of the transposon insertion for Tam1 in *cyc-608*, and Ram1 in *rad-609*. The oligos pairs were gttcttgagtccaccgctttgttc and aatgccgatggataaacggactct for *CYC*, caccggtggtaacatgaaaactgac and tgcttgctatgtgattgaacaaaacc for *RAD*, ggccgactacaatatccagaaggag and gaaccgaaccatcagacaaacaaac for *AmUBI*. PCR programmes were as follow: initial denaturation at 95°C 2 min; 40 cycles of 95°C 15 s; 55°C (*CYC*), 58°C (*RAD*), 60°C (*AmUBI*) 30 s; 72°C 30 s; and 72°C 10 min, after which melting curve were recorded from 70°C to 95°C, every 0.5°C. PCR reactions were performed in quadruplicate in an Opticon real-time PCR instrument (MJ Research), using SYBR Green JumpStart (Sigma). *Ct* values were obtained with a threshold of 0.105 using the software Opticon Monitor 3.1 (MJ Geneworks).

### Lines with Combinations of *CYC* and *RAD* Activity

Lines with combinations of *CYC* and *RAD* wild-type and mutant activities were obtained from by crossing single mutants *cyc-608* (JI:608) and *rad-609* (JI:609) and the double mutant *cyc-608 rad-609* (JI:727) to the wild type. Genotyping for the *CYC^maj^* wild-type allele was done using the oligos tcctcccttcactctcgcgc and tggcgcatagctggttcgac, spanning most of the coding region (annealing temperature 55°C). Presence of *CYC^maj^* wild-type allele gave a PCR product of 790 bp. Genotyping for the *cyc-608* mutant allele was done using the oligos tcctcccttcactctcgcgc and gtgacccatgcactcttgg spanning from the coding region to the Tam4 transposon insertion (annealing temperature of 57°C). Presence of *cyc-608* mutant allele gave a PCR product of 327 bp. Genotyping for the *RAD^maj^* wild-type allele was done using the oligos tccaacaagaccttttgattcc and tgcttcgtttattatctgaacgatt, spanning from the 5′UTR to the 3′UTR, including the two exons and the intron (annealing temperature 55°C). Presence of the *RAD^maj^* wild-type allele gave a PCR product of 1,452 bp. Genotyping of the *rad-609* mutant allele was done using the oligos tccaacaagaccttttgattcc and taaggaagcttcgggtccgg spanning from the 5′UTR towards the first exon, part of the intron, and the Ram1-like insertion (annealing temperature 60°C). Presence of the *rad-609* mutant allele gave a PCR product of ∼1 kb.

Based on *CYC^cha^* and *RAD^cha^* sequences obtained from *A. charidemi*, CAPS markers were designed. For *CYC*, A PCR product of 791 bp on the coding region was obtained using the oligos tcctcccttcactctcgcgc and tggcgcatagctggttcgac. The PCR product was digested with the restriction enzyme KpnI (Invitrogen) generating two fragments in the *CYC^maj^* allele (675 bp and 116 bp), but did not digest *CYC^cha^* allele. For *RAD*, a PCR product of 796 bp, covering the second exon and further on down-stream the *RAD* stop codon, was obtained using the oligos tgcatgcaggttcagaaatc and tttgggctatttcgcttgac. The PCR product was digested with the restriction enzyme AluI (Roche) producing two fragments on *RAD^maj^* allele (444 bp and 352 bp), and three fragments on *RAD^cha^* allele (444 bp, 200 bp, and 152 bp).

### Generation of Lines Introgressed with *CYC* and *RAD* Alleles

A collection of BC3 and SBC3 plants carrying *CYC^cha^ RAD^cha^* were screened using the CAPS markers. A line with genotype *CYC^maj^RAD^maj^/CYC^cha^RAD^cha^* was selected and further backcrossed. The BC4 population of plants carrying *CYC^cha^* and *RAD^cha^* was screened with AFLP markers to select the one with the most homogeneous *A. majus* genome. This plant was backcrossed to generate a BC5, the progeny of which was in turn used for morphological and gene expression analysis.

### GEM Surface Fitting

The GEM space was smoothed by fitting a 2-D function to the data. The fitting was achieved using the least-square method [55]. The function was performed using the MATLAB function fminsearch, which finds a local maximum depending on given initial parameter values. The function fitted for the GEM space was:
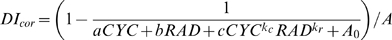


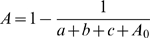
where *a*, *b*, *c*, *k*_c_, *k*_r_, and *A*_0_ are the parameters of the function (Table 2), *CYC* and *RAD* are the gene expression levels. The parameter *A* has been fixed to ensure that the wild-type genotype (i.e., *CYC* = 1 and *RAD* = 1) has a *DI*_cor_ = 1.

**Table 2:** Parameters of fitted function for the GEM space (*R* ^2^ = 0.263; explained variance = 0.816).

Parameter	Fitted Value
*k*_c_	0.034
*k*_r_	0.020
*A*_0_	1.159
*a*	−0.007
*b*	−0.011
*c*	−0.261

### Estimation of Drift Load

For a variety of models of selection, the expected loss of fitness due to drift around the optimum is ∼1/4*N*_e_ for each degree of freedom, independent of the selection strength: strong selection leads to smaller fluctuations, so that the net effect on fitness is the same as with weaker selection. This result arises from Wright's formula [56] for the stationary distribution under mutation, selection, and drift, which shows that the trait distribution is 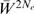 multiplied by the distribution in the absence of selection. The argument applies to stabilising selection on multiple polygenic traits, or to small fluctuations in allele frequency at balanced polymorphisms; if frequency-dependent selection maintains polymorphism, then the drift load is typically twice as large [57]. Matters are more complicated when variation is maintained by a balance between mutation and selection. If we focus on a single locus, the outcome depends on how mutation acts. With a continuum of allelic effects, drift has little overall effect on the mean fitness: though it reduces fitness by causing fluctuations in the mean around the optimum, which is counterbalanced by a reduction in variance. Nevertheless, there is still heterosis of ∼1/8*N*_e_ per locus, because an F1 individual is on average closer to the optimum. If variation involves rare deleterious alleles, then the drift load, and hence the heterosis, are smaller, in proportion to the frequency of deleterious alleles. In this case, heterosis is contributed only by those loci with 4*N*_e_ *s*≈1, for which selection and drift have comparable strength. It is this latter case, in which weakly deleterious alleles can be fixed by drift, that has previously been discussed [58]–[59]. Detailed derivations are given in Text S1 and Figure S2.

## Supporting Information

Figure S1 **The notch phenotype in double heterozygote F1 hybrids.** Left, hybrids with “notch” phenotype. Right, hybrids with wild-type phenotype. Grey line denotes the “notch.”
(1.56 MB TIF)

Figure S2
**The effect of random drift on the mutation load at a biallelic locus.** The mutation load, relative to its maximum *s*, is plotted against *N*_e_ *s*. The dashed lines at the right show the large *N*_e_ *s* limit with no dominance (heavy lines) and for *h* = 0.05 (light dashed lines). The dashed line at the left shows the limit of small *N*_e_μ, which is independent of dominance: 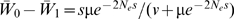. In all cases, backmutation is rare (*v* = μ/10). The upper heavy curve is for 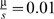, 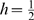; as *N*_e_ *s* decreases, the load increases from 2μ (dashed line at right) to its maximum, *s*, at left. The light line below is for *h* = 0.05; now, the deterministic load is slightly lower.(0.37 MB TIF)

Text S1 **Heterosis and the drift load.**
(0.14 MB DOC)

## Abbreviations

BC: backcross
df: degree of freedom
CAPS: cleaved amplified polymorphic sequences
DI: dorsalisation index
GEF: gene expression fitness
GEM: gene expression morphology
PC: principal component
RT-PCR: reverse transcription PCR
