# Supplementary figures and images for: Cryptic Variation between Species and the Basis of Hybrid Performance
Source: PLoS Biol. 2010 Jul 20;8(7):e1000429. doi: 10.1371/journal.pbio.1000429 (PMC2907293; doi:10.1371/journal.pbio.1000429)

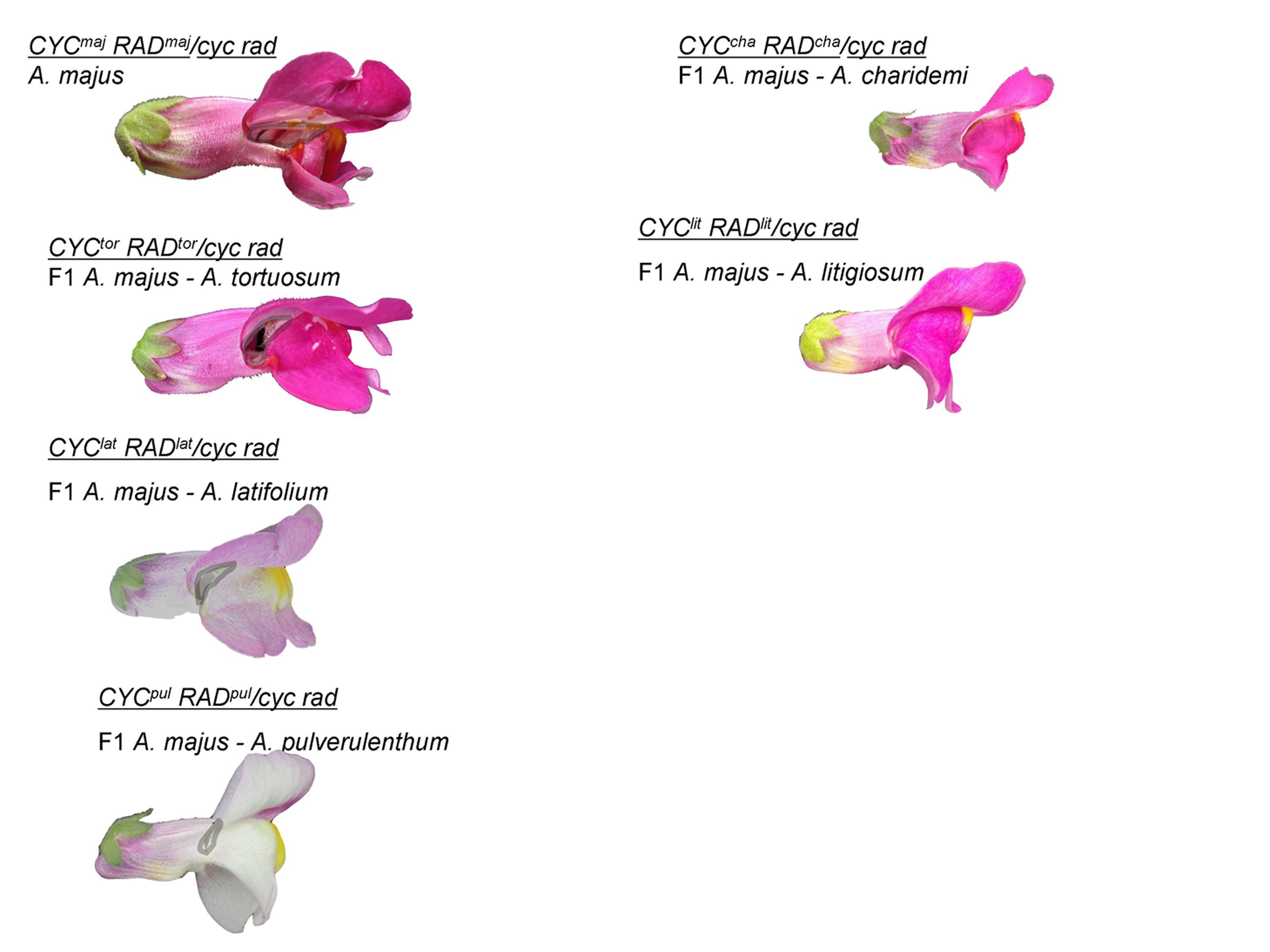

Supplement: Figure S1 — The notch phenotype in double heterozygote F1 hybrids. Left, hybrids with “notch” phenotype. Right, hybrids with wild-type phenotype. Grey line denotes the “notch.” (1.56 MB TIF) [file pbio.1000429.s001.tif]

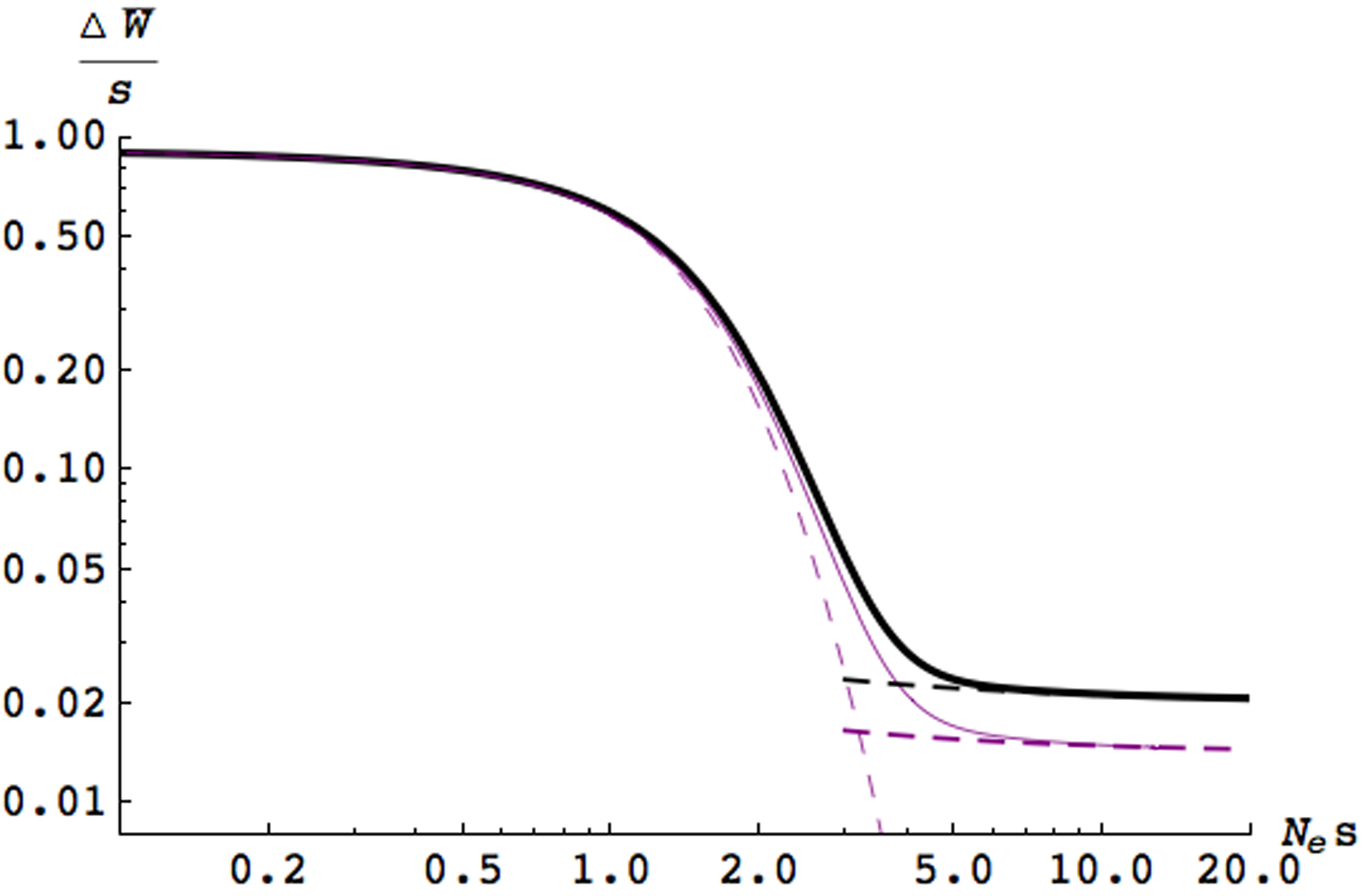

Supplement: Figure S2 — The effect of random drift on the mutation load at a biallelic locus. The mutation load, relative to its maximum s, is plotted against N e s. The dashed lines at the right show the large N e s limit with no dominance (heavy lines) and for h = 0.05 (light dashed lines). The dashed line at the left shows the limit of small N eμ, which is independent of dominance: . In all cases, backmutation is rare (v = μ/10). The upper heavy curve is for , ; as N e s decreases, the load increases from 2μ (dashed line at right) to its maximum, s, at left. The light line below is for h = 0.05; now, the deterministic load is slightly lower. (0.37 MB TIF) [file pbio.1000429.s002.tif]
