# Supplementary material for: Cryptic Variation between Species and the Basis of Hybrid Performance
Source: PLoS Biol. 2010 Jul 20;8(7):e1000429. doi: 10.1371/journal.pbio.1000429 (PMC2907293; doi:10.1371/journal.pbio.1000429)
Supplement: Text S1 — Heterosis and the drift load. (0.14 MB DOC) [file pbio.1000429.s003.doc]

**Text S1. Heterosis and the drift load**

**(Rosas, Barton *et al*; Cryptic variation and hybrid performance)**

**Heterosis in the F1 and F2**

We first consider the magnitude of heterosis as a function of the allele frequencies, without specifying what caused divergence. This approach parallels Whitlock et al. (2000), who consider how the drift load and heterosis depend on *FST* in a structured population, assuming biallelic loci. Imagine a metapopulation with a distribution of allele frequencies, which averages $\bar{p}$; let $\bar{q}=1-\bar{p}$. Two populations are sampled, with allele frequencies *p*_1_, *p*_2_. We calculate the mean fitness, $\bar{W}_{0}$, of a population at the mean, $\bar{p}$; of a population sampled at random, $\bar{W}_{1}$; of an F1 between two randomly sampled populations, $\bar{W}_{F1}$; and from a population at Hardy-Weinberg and linkage equilibrium, derived from such a cross, $\bar{W}_{F2}$ (strictly speaking, this will only equal the F2 fitness if there is no linkage). Assuming two alleles, and genotypic fitnesses 1: 1 - *hs*: 1 - *s*, these fitnesses are:

[1]

If deleterious alleles tend to be recessive (*h* < 1 /2), then random drift reduces mean fitness. In the F1, the mean fitness is restored to that of a population at the overall mean, whilst in the F2, half of that heterosis is lost. (Note that Whitlock et al., 2000, measure heterosis as ; the heterosis in the F1 would be twice as great). We also consider a continuum-of-alleles model, in which each allele has a value *y*, and the fitness of a diploid individual with allelic values {*y*, *y**} is $1-\frac{s}{2}z^{2}$, where the diploid phenotype is *z* = *y* + *y**. A population is characterised by its mean and variance, $\bar{z}$, *v* = var(*z*), and ; the overall mean is assumed to be at the optimum, $E[\bar{z}]=0$

Then:

$$\bar{W}_{0}=1-\frac{s}{2}v*$$

[2]

The baseline, $\bar{W}_{0}$, is assumed to be a population at the deterministic equilibrium, with variance *v** maintained by mutation/selection balance. With both two alleles, and a continuum of alleles, half the heterosis is lost when the F1 segregates out to produce a hybrid F2 population at Hardy-Weinberg. However, there is a different relation between heterosis in the F1 and the drift load in the two cases. With two alleles, the heterosis in the F1 equals the drift load , whereas with a continuum-of-alleles, the heterosis in the F1 is half the component of the drift load that is due to fluctuations in the mean . However, to the extent that random drift reduces the variance of allelic effects $(E[v]<v*)$, the total drift load will be *less* than expected from fluctuations in the mean .

**Divergence under selection and drift**

Populations may diverge for many reasons, but we focus on random sampling drift in a uniform evironment. (Heterogeneous selection could produce much stronger divergence in allele frequencies, but the consequent heterosis would be offset by local adaptation, which reduces hybrid fitness). We first consider polymorphism under selection and drift, and then the more complex case of a mutation/selection/drift balance. When mutation is negligible, and genotypic fitnesses are fixed, the distribution of allele frequencies at a locus is proportional to $\bar{W}^{2N_{e}}$. Under strong overdominant selection, allele frequencies cluster sharply around the adaptive peak. With two alleles, expanding around the maximum of $\bar{W}$ gives a distribution with variance , and so mean fitness is reduced by an average of . This argument extends to *k* alleles, giving a drift load $(k-1)/(4N_{e})$, or $1/(4N_{e})$ per degree of freedom. If polymorphism is instead maintained by frequency-dependent selection, then Wright's distribution no longer holds. However, it is still possible to find the effect of small fluctuations on mean fitness. Kimura and Ohta (1970) showed that (assuming no dominance) the drift load is twice as great as with overdominance, at $1/(2N_{e})$per degree of freedom.

The same argument applies to a polygenic trait under stabilising selection, provided that we assume a fixed genetic variance. Again, the distribution of the trait mean is distributed as $\bar{W}^{2N_{e}}$ (Lande, 1976), and so the loss of fitness due to fluctuations around the mean is $1/(4N_{e})$ per degree of freedom. We discuss below what the relevant number of "degrees of freedom" might be.

So, for either overdominant selection on allele frequencies, or stabilising selection on the mean of a quantitative trait, the mean fitness is reduced by $1/(4N_{e})$ per degree of freedom as a result of small fluctuations around the equilibrium. In both cases, an F1 hybrid between two independently evolved populations will have a mean value that is raised by half this drift load (Eqs. [1], [2]).

**Divergence under mutation, selection and drift**

If variation is maintained by deleterious mutations, then the magnitude of the drift load, and its relation with heterosis, depend on the strength of selection relative to drift, and on the genetic model. With discrete alleles, drift load and heterosis are very small unless $N_{e}s\sim1$, whereas with a continuum of allelic effects, heterosis depends primarily on $1/4N_{e}$, as in the absence of mutation, discussed above.

Discrete alleles

Assume a mutation rate to deleterious alleles of *µ* per locus, and *v* < *µ* in the reverse direction; fitnesses are 1: 1 - *hs* : 1 - *s*, as above. For $N_{e}\mu<<1$, loci are usually fixed, and the probability of fixation of the deleterious allele is $\mu e^{-2N_{e}s}/(v+\mu e^{-2N_{e}s})$; hence, the drift load is just , independent of the dominance, *h*. An F1 would have fitness increased by . Thus, heterosis is still closely related to the drift load, but both will be small if $N_{e}s$ is large. If $N_{e}\mu\sim1$, so that there is typically some polymorphism, the mean fitness can be calculated numerically from Wright's distribution (Whitlock et al., 2000). We can find an approximation for large $N_{e}s$by considering the expected change in allele frequency. With no dominance $\left(h=\frac{1}{2}\right)$, and ignoring back-mutation (*v*<<*µ*):

$E[\Delta p]=0=\mu\bar{q}-E\left[\frac{s}{2}pq\right]$ [3]

Therefore, $\bar{p}=\frac{2\mu}{s(1-F)}$, and the mutation load is increased by a factor 1/(1-*F*). This is because drift reduces the expected heterozygosity, and hence the efficiency of selection. For large $N_{e}s$ and $\bar{p}<<1$ we have $F\sim1/2N_{e}s$, and so the load is increased by $\sim\frac{\mu}{N_{e}s}$ per locus. With partially recessive alleles, the calculation is more involved (see Whitlock, 2002, who makes a similar calculation for the island model with migration). However, numerical calculations suggest that as long as *µ* << *s*, and $h>\sqrt{\frac{\mu}{s}}$, so that mutations are eliminated in heterozygotes, dominance has little effect, and the load is still increased by a factor $\frac{1}{1-F}\sim1+\frac{1}{2N_{e}s}$. Fig S2 shows that the two approximations - that loci are near fixation, or that *N_e_s* is large - are accurate over most of the range of *N_e_s*.

A continuum of alleles

Now, consider the continuum-of-alleles model, with mutation occurring at rate *µ*, and changing the value of the allele by a random Gaussian with mean zero, variance *σ^2^*. The increase in trait variance due to mutation at one locus is $v_{m}=\mu\sigma^{2}$. First, consider the deterministic limit. If new mutations have variance much smaller than the standing variance (*σ^2^* << *v*), then the distribution of allelic effects is approximately Gaussian, and $v=\sqrt{V_{m}/s}$ (Lande, 1976). However, if new mutations have large effects (*σ^2^* >> *v*), then allelic effects follow a leptokurtic distribution. Under the House of Cards approximation, in which new mutations have value independent of their parent, *v* = 2 *µ*/*s* (Turelli, 1984). With random drift, the loss of mean fitness due to fluctuations around the optimum is $\frac{1}{4N_{e}}$, whatever the distribution of allelic effects (Bürger, 2000, Eq. 2.14); this is true both for a single haploid locus, and for a quantitative trait. However, drift also reduces the genetic variance, which increases the mean fitness. Bürger (2000, p. 268 ff) gives approximations for the effect of drift on the genetic variance under both the Gaussian regime, and the House of Cards approximations, for a quantitative trait in diploids, that depends on *n* loci. Under the Gaussian approximation, Bürger's (2000) Eq. 2.5 shows that to leading order in 1/*N_e_*, drift reduces genetic variance by $\frac{n}{2N_{e}s}$, and hence increases mean fitness by $\frac{n}{4N_{e}}$.

Thus, random drift *reduces* the mutation load due to stabilising selection on a polygenic trait: the reduced variance at *n* loci outweighs the effect of fluctuations in the trait mean. For stabilising selection on a *single* diploid locus, however, the effects cancel, and drift has no effect on mean fitness, to order 1/*N_e_*.

When mutations have large effects, the House-of-Cards approximation is the harmonic mean of the predictions at the extremes of strong and weak selection: $v\sim1/\left(1/\left(\frac{4n\mu}{s}\right)+1/(4N_{e}nv_{m})\right)$. (We write the total mutational variance *V_m_*=2*nv_m_* in

terms of the mutational variance per gene, *v_m_*=*µσ*^2^). To leading order, the genetic

variance and the mutation load are reduced by a factor $\left(1-1/(N_{e}s\sigma^{2})\right)$. Note that the selection coefficient against a new mutation averages *sσ*^2^*/* 2, and so as with discrete alleles, drift will have a negligible effect unless this selection is of order 1/*N_e_*. Our main concern is with the strength of heterosis. Since this depends only on fluctuations in the mean, and not on the genetic variance maintained at each locus, this is still equal to half the drift load due to fluctuations in the mean, 1/8*N_e_*.

**References Text S1**

Burger, R. 2000. The mathematical theory of selection, recombination and mutation. Wiley, Chichester.

Kimura, M., and T. Ohta. 1970. Genetic loads at a polymorphic locus maintained by frequency dependent selection. Genet.Res. 16 : 145 - 150.

Lande, R. 1976. Natural selection and random genetic drift in phenotypic evolution. Evolution 30 : 314 - 334.

Turelli, M. 1984. Heritable genetic variation via mutation - selection balance : Lerch' s zeta meets the abdominal bristle. Theoretical Population Biology 25 : 138 - 193.

Whitlock, M. C. 2000. Fixation of new alleles and the extinction of small populations: drift load, beneficial alleles, and sexual selection. Evolution 54 : 1855 - 1861.

Whitlock, M. C., P. K. Ingvarsson, and T. Hatfield. 2000. Local drift load and the

heterosis of interconnected populations. Heredity 84 : 452 - 457.

Whitlock, M. C. 2002. Selection, load and inbreeding depression in a large

metapopulation. Genetics.
